# Attitudes toward psychedelics and psychedelic-assisted psychotherapy among Australian mental healthcare providers

**DOI:** 10.1177/00048674251346679

**Published:** 2025-07-15

**Authors:** Zohaib Nadeem, Stephen Parker, Hugh McGovern, Bianca Sebben, Lena KL Oestreich

**Affiliations:** 1 Faculty of Medicine, The University of Queensland, Brisbane, QLD, Australia; 2 Metro North Mental Health, Royal Brisbane and Women’s Hospital, Herston, QLD, Australia; 3 Faculty of Medicine, Griffith University, Brisbane, QLD, Australia; 4 IMPACT – The Institute for Mental and Physical Health and Clinical Translation, School of Medicine, Barwon Health, Deakin University, Geelong, VIC, Australia; 5 Queensland Institute of Medical Research (QIMR) Berghofer, Brisbane, QLD, Australia; 6 School of Psychology, The University of Queensland, Brisbane, QLD, Australia; 7 Centre for Advanced Imaging (CAI) and Australian Institute for Bioengineering and Nanotechnology (AIBN), The University of Queensland, Brisbane, QLD, Australia

**Keywords:** Psychedelics, psilocybin, MDMA, Australia, psychiatrists, psychologists

## Abstract

**Background:**

Recent regulatory changes in Australia have approved 3,4-methylenedioxymethamphetamine for treating post-traumatic stress disorder and psilocybin for treatment-resistant depression. However, limited data exists on Australian mental healthcare providers’ attitudes, knowledge and readiness to implement with psychedelic-assisted psychotherapy.

**Methods:**

A cross-sectional survey was conducted between December 2023 and March 2024, targeting Australian general practitioners, psychiatrists and psychologists. Participants completed online questionnaires developed based on the Knowledge, Attitude and Practices framework to explore mental healthcare providers’ knowledge, attitudes and practices toward psychedelics.

**Results:**

The survey was completed by 109 clinicians (21% psychiatrists, 56% psychologists, 23% general practitioners). Attitudes toward psychedelic-assisted therapy were positive. However, safety and efficacy concerns persisted, particularly among psychiatrists, who were significantly more likely than psychologists to perceive psychedelic use as unsafe under medical supervision and question the scientific rigor of current research. Self-rated knowledge positively predicted actual knowledge, though many clinicians relied on informal sources such as podcasts and Internet-based media, highlighting gaps in evidence-based education. Clinicians with personal experience of psychedelic use expressed higher levels of agreement with statements relating to psychedelics improving outcomes in conjunction with psychotherapy and showing promise in treating psychiatric disorders.

**Conclusion:**

While Australian mental healthcare providers generally support psychedelic-assisted psychotherapy, significant safety and efficacy concerns remain, particularly among psychiatrists. Targeted educational initiatives from professional bodies, emphasizing evidence-based training and accessible resources, are essential to support informed clinical decision-making and safe therapeutic practices in this emerging field.

## Introduction

Psychedelics are substances with hallucinogenic and consciousness-altering properties, primarily due to their serotonergic agonism at 5-HT2A receptors (Nichols, 2016). Classic psychedelics include lysergic acid diethylamide (LSD), psilocybin, dimethyltryptamine (DMT), mescaline, peyote and ayahuasca (Nichols, 2016). Entactogens like 3,4-methylenedioxymethamphetamine (MDMA) also exhibit psychedelic-like properties but have a different underlying mechanism of action (Nichols, 2016; Sarparast et al., 2022). In recent years, there has been a resurgence of interest in the potential therapeutic applications of psychedelics for psychiatric disorders and symptoms, including post-traumatic stress disorder (PTSD), end-of-life anxiety, major depressive disorder (MDD), obsessive compulsive disorder (OCD), anxiety and substance use disorders (Nichols, 2016; Rucker et al., 2018). Several clinical trials have bolstered this enthusiasm, including clinical trials of MDMA for PTSD (Mitchell et al., 2023; Ot’alora G et al., 2018; Ponte et al., 2021), and several randomized control trials (RCTs) supporting the efficacy of psilocybin in treating MDD (Carhart-Harris et al., 2021; Goodwin et al., 2022; Haikazian et al., 2023; Raison et al., 2023; von Rotz et al., 2023).

In light of the emerging evidence base, the Australian Therapeutic Goods Administration (TGA) approved the use of MDMA for PTSD and psilocybin for treatment-resistant MDD in 2023 (Administration (TGA) TG, 2023). These substances were reclassified from Schedule 9 (i.e. prohibited substances) to Schedule 8 (i.e. controlled substances), permitting prescription by psychiatrists with special approval. Conversely, shortly after this change in Australia, the United States Food and Drug Administration (FDA) denied approval of MDMA for the treatment of PTSD (Reardon, 2024). Specifically, scientists on the FDA advisory committee raised concerns about inadequate placebo controls, prior MDMA use among participants, additional treatments sought during follow-up and the confounding effects of psychotherapy. Although the FDA’s formal rationale has not been publicly released, as such communications are sent privately to the sponsor, these concerns likely informed the agency’s decision. Despite the uncertainty posed by this regulatory decision, researchers have proposed strategies for addressing these concerns while several studies continue to investigate the therapeutic potential of psilocybin, DMT and LSD (Hartstein and Menon, 2024; Heidi Anne Duerr, 2024; Roseman, 2024; Wolfgang et al., 2025).

The TGA approval of MDMA and psilocybin in Australia was not without controversy. Critics argue that the approval decision was rushed, with insufficient input from experts in the field, limited evidence of efficacy and inadequate safety data (Nogrady, 2023; Rossell et al., 2023). Additional concerns have been raised regarding unclear protocols for practitioners and a lack of oversight to ensure equitable access across Australia (Haridy, 2023; Kisely, 2023). In contrast, a recent study published by our group found that the general Australian public had positive attitudes toward psychedelic-assisted psychotherapy and further related research (Nadeem et al., 2024). However, concerns about safety and efficacy persisted among respondents, along with notable misconceptions and knowledge gaps.

While mainstream attention has focused on a select group of experts in the field, little is known about the attitudes and perceptions of Australian mental health professionals generally. Surveys with Australian psychiatrists and psychiatry registrars prior to the TGA approval demonstrated enthusiastic support for psychedelic-assisted psychotherapy, with acknowledgement of safety concerns, limited efficacy data and personal knowledge gaps (Berger and Fitzgerald, 2023; Grover et al., 2023). Similarly, a 2022 qualitative study found Australian political stakeholders expressed cautious optimism toward psychedelic-assisted psychotherapy, but hesitated to offer public support due to limited evidence and persisting stigma (Kunstler et al., 2023). However, since the TGA approval decision in February 2023, there has been no formal re-evaluation of the attitudes, perceptions and knowledge base of Australian mental health practitioners regarding psychedelics and psychedelic-assisted psychotherapy. To address this gap, we surveyed the attitudes, knowledge, readiness and personal experiences of healthcare practitioner groups with the potential to be involved in referral and administration of psychedelic-assisted therapy in Australia, namely psychiatrists, psychologists and general practitioners (GPs).

## Methods

We implemented a cross-sectional survey. Ethics approval was granted by the University of Queensland Human Research Ethics Committee A (Application ID: 2007718). The Methods and Results are reported in accordance with the Checklist for Reporting Survey Studies (Sharma et al., 2021) (see Supplementary Material 1).

### Participants

Recruitment targeted GPs, Psychologists and Psychiatrists in Australia. Convenience sampling was used and facilitated through advertisements at professional organizations, professional networks and snowball sampling. To prevent multiple submissions from the same device, the Qualtrics setting ‘Prevent multiple submissions’ was enabled. No direct compensation was provided, but clinicians had the option to enter a draw to win a $150 gift card as an acknowledgement of their participation. The recruitment window was open between December 2023 and March 2024. The target sample size was a minimum of 37 participants per healthcare profession (total *n* = 111). This sample size was determined to allow for analysis of variance (ANOVA) with a medium effect size (Cohen’s *d* = 0.5), a power of 0.8 and an alpha level of 0.05.

### Design and questionnaires

Participants provided informed consent online before completing questionnaires on the online platform Qualtrics via a mobile device or a computer. Demographics, including age, gender, sexual orientation, race/ethnicity, religion, professional degree, years in practice, setting and specialty area, were recorded.

To assess knowledge, attitudes and clinical practices regarding the therapeutic use of psychedelics, we used a questionnaire developed by Kucsera et al. (2023). This survey was designed to capture the depth of healthcare professionals’ knowledge about psychedelic-assisted therapy, their personal and professional attitudes toward its use, and details regarding related clinical practices (Barnett et al., 2018, 2022). The first section assessed ‘knowledge’ and considered respondents’ understanding of various aspects of psychedelic-assisted therapy, including mechanisms of action, therapeutic protocols, potential benefits and associated risks. Respondents indicated their knowledge level on a 10-point Likert-type scale ranging from 0 (none) to 10 (high). The second section explored ‘attitudes’, measuring respondents’ perspectives on the acceptability of psychedelics in therapeutic settings, ethical considerations and their views about psychedelic therapies. Items in this section were rated on a 7-point Likert-type scale from 1 (strongly disagree) to 7 (strongly agree). Finally, the third section considered ‘clinical practices’ regarding the respondents’ experience, if any, with psychedelic therapy. This included current practices, willingness to refer patients for such treatments and perceived barriers to integrating psychedelics into practice. This section used a 5-point Likert-type scale, where participants indicated the frequency of engagement or the likelihood of participating in specific clinical practices, with options ranging from 1 (never) to 5 (always). Additional questions about the legality of psychedelics and psychedelic-assisted therapy in Australia were included. For the full adaptation of this survey, please see Supplemental Material 2. Finally, we used a questionnaire developed by Corrigan et al. (2022) to examine factors related to psychedelic substance use, psychedelic therapies and general substance use (Corrigan et al., 2022). This tool includes questions rated on a 5-point Likert-type scale, with response options ranging from 1 (strongly disagree) to 5 (strongly agree).

### Data availability statement

The data used in this study are available from the corresponding author upon request.

### Data analysis

All statistical analyses were performed using SPSS Statistics, version 29.0.1.0 (IBM SPSS Statistics for Macintosh, 2023) and RStudio 2023.12.0 (RStudio: Integrated Development Environment for R, 2023). Given the sample size and the use of composite scores for several measures, parametric tests were primarily employed. Chi-square tests were conducted to compare categorical demographic and clinical variables across clinician groups. Responses for ‘prefer not to say’ were included in these descriptive analyses. For all subsequent analyses related to knowledge and attitudes toward psychedelics and psychedelic-assisted therapy, participants who skipped the question or indicated ‘prefer not to say’ were excluded from the analysis.

Group comparisons of continuous demographic and clinical data were conducted using ANOVA. For categorical data, Chi-square tests were applied. Post hoc comparisons were corrected using Bonferroni correction. The Wilson score method was used to analyze proportions to test whether the observed proportion of correct responses significantly differed from alternative responses. To test whether perceived knowledge was associated with accurate legal knowledge, logistic regression was conducted with predictors including perceived knowledge of the benefits, risks, side effects and therapeutic applications of psychedelics and the outcome variable’s actual knowledge about the current legal status of psychedelics in Australia’. A multiple linear regression analysis, using the same predictors and the outcome variable ‘willingness to provide preparation and/or integration therapy for clients who have taken psychedelic medicine independently’, was used to assess clinicians’ sense of being adequately informed and their willingness to provide psychedelic-related services. Finally, logistic regression analyses were conducted to evaluate the association between personal experience with psychedelics and clinicians’ agreement with the therapeutic potential of psychedelics in mental health care, as well as their willingness to provide services related to psychedelic-assisted therapies. For all regression analyses with multiple predictors, variance inflation factors (VIF) were calculated for all predictors to confirm that multicollinearity was within acceptable limits (VIF < 5).

## Results

A total of 169 respondents commenced the survey. Sixty were excluded due to incomplete responses or occupations other than GP, psychiatry, or psychology, resulting in a final sample size of 109 participants for analysis. Most respondents resided in Queensland (52.3%), were aged between 25 and 44 (53.2%), identified as Caucasian (58.7%), female (57.8%), heterosexual (69.7%) and reported no religious affiliation (52.3%). Psychologists (including provisional psychologists) made up most of the sample (56%), compared to Psychiatrists (including psychiatry registrars) (21%) and GPs (23%). Almost half of the sample worked in a private practice setting (45%). For a detailed overview of demographic variables and information by clinician groups, see Table 1.

**Table 1.** Demographics and clinical experience.

	Psychologists (*n* = 61)	Psychiatrists (*n* = 23)	General Practitioners (*n* = 25)	
*Demographic information*				
Age (years), *n* (%)				χ^2^(14) = 46.04, *p* **<** **0.001**
18–24	6 (10%)	0 (0%)	1 (4%)	
25–34	22 (36%)	3 (13%)	4 (16%)	
35–44	16 (26%)	5 (22%)	8 (32%)	
45–54	13 (21%)	8 (35%)	6 (24%)	
55–64	1 (2%)	6 (26%)	4 (16%)	
65+	3 (5%)	1 (4%)	2 (8%)	
Gender identity, *n* (%)				χ^2^(8) = 11.39, *p* = 0.181
Female	40 (66%)	10 (43%)	13 (52%)	
Male	16 (26%)	8 (35%)	6 (24%)	
Queer	0 (0%)	1 (4%)	0 (0%)	
Gender fluid	1 (2%)	0 (0%)	0 (0%)	
Prefer not to say	4 (7%)	4 (17%)	6 (24%)	
Sexual orientation, *n* (%)				χ^2^(12) = 11.32, *p* = 0.501
Heterosexual	45 (74%)	14 (61%)	17 (68%)	
Bisexual	5 (8%)	1 (4%)	0 (0%)	
Homosexual	1 (2%)	1 (4%)	0 (0%)	
Pansexual	2 (3%)	1 (4%)	1 (4%)	
Queer	1 (2%)	0 (0%)	0 (0%)	
Prefer not to say	7 (11%)	6 (26%)	7 (28%)	
Race/ethnic identity, *n* (%)				χ^2^(22) = 30.59, *p* = 0.105
Caucasian	42 (69%)	12 (52%)	10 (40%)	
Australian	10 (16%)	1 (4%)	3 (12%)	
East Asian	0 (0%)	2 (9%)	3 (12%)	
Other^a^	9 (15%)	8 (35%)	9 (36%)	
State/Territory, *n* (%)				χ^2^(14) = 46.1, *p* **<** **0.001**
Queensland	43 (70%)	6 (26%)	8 (32%)	
Victoria	6 (9%)	5 (22%)	4 (16%)	
New South Wales	9 (15%)	7 (11%)	4 (16%)	
South Australia	1 (2%)	0 (0%)	1 (4%)	
Western Australia	1 (2%)	0 (0%)	5 (20%)	
Australian Capital Territory	0 (0%)	3 (13%)	0 (0%)	
Tasmania	0 (0%)	1 (4%)	3 (12%)	
Prefer not to say	1 (2%)	1 (4%)	0 (0%)	
Religious affiliation, *n* (%)				χ^2^(16) = 26.36, *p* **=** **0.049**
Christian	14 (23%)	4 (17%)	7 (28%)	
Spiritual	1 (2%)	0 (0%)	3 (12%)	
None	36 (59%)	10 (43%)	11 (44%)	
Other^b^	6 (9%)	5 (23%)	0 (0%)	
Prefer not to say	4 (7%)	4 (17%)	4 (16%)	
*Clinical experience*				
Years in clinical practice, *n* (%)				χ^2^(16) = 41.7, *p* **<** **0.001**
0–5	28 (46%)	1 (4%)	1 (4%)	
6–10	7 (11%)	2 (9%)	2 (8%)	
11–15	13 (21%)	3 (13%)	8 (32%)	
16–20	5 (8%)	3 (13%)	2 (8%)	
21–30	1 (2%)	5 (22%)	3 (12%)	
>30	2 (3%)	5 (22%)	5 (22%)	
Prefer not to say	5 (8%)	2 (9%)	2 (8%)	
Clinical setting, *n* (%)				χ^2^(14) = 39.05, *p* **<** **0.001**
Private practice (outpatient)	27 (44%)	3 (13%)	19 (76%)	
Public community	13 (21%)	10 (43%)	2 (8%)	
Public hospital	2 (3%)	3 (13%)	0 (0%)	
University/ education	10 (16%)	0 (0%)	0 (0%)	
Other^c^	4 (7%)	2 (9%)	0 (0%)	
Prefer not to say	5 (8%)	5 (22%)	4 (16%)	

NGO = Non-governmental organization.

a Other = Indigenous, East Asian, South Asian, Southeast, Asian, Fijian, Black, Caribbean, Middle Eastern, Jewish, Prefer not to say.

b Other = Hindu, Buddhist, Jewish, Pagan, Animism.

c Other = Private hospital, NGO, Correctional center.

### Legal status of psychedelics in Australia

#### Overall sample

**Figure 1.**
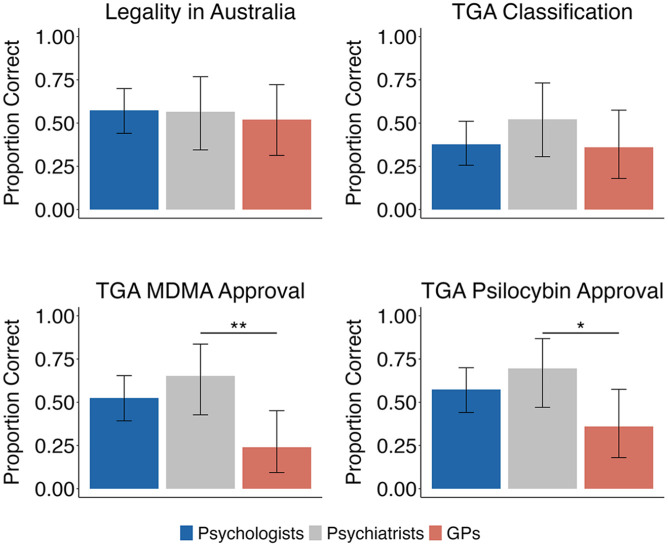
Knowledge of the legal status and Therapeutic Goods Administration (TGA) classification and approvals of psychedelics in Australia. Violin plots display the distribution of responses for each group. Error bars represent the standard error of the mean. MDMA = 3,4-methylenedioxymethamphetamine; GP = general practitioner. *p < 0.05, **p < 0.01, ***p < 0.001.

There were no significant differences in the levels of knowledge regarding the current legal status of psychedelics among clinicians. While 67% of respondents correctly identified that psychedelics are approved exclusively for medical use in Australia (*Z* = 3.25, *p* = 0.001), only 47% (non-significant) correctly identified psychedelics as Schedule 8 (Controlled Substances, see Figure 1). A non-significant proportion of participants correctly identified that MDMA had been approved by the TGA for the management of PTSD (57%). In comparison, 64.5% correctly identified that psilocybin had been approved for the management of treatment-resistant depression (*Z* = 2.8, *p* = 0.001).

#### Group comparisons

Significantly more psychiatrists (79%) than GPs (30%) correctly identified MDMA as approved for the treatment of PTSD (*Z* = 3.53, *p* = 0.004, Bonferroni corrected) and psilocybin (psychiatrists = 84%, GPs = 45%) for the management of treatment-resistant depression (*Z* = 2.8, *p* = 0.022, Bonferroni corrected).

### Self-rated knowledge

#### Overall sample

Most clinicians felt they had a good understanding of the side effects (*M* = 6.33, *SD* = 2.79), risks (*M* = 6.36, *SD* = 2.68) and benefits of psychedelics (*M* = 6.71, *SD* = 2.59), including their therapeutic applications (*M* = 6.64, *SD* = 2.53). While the majority (79.1%) of clinicians felt it was very important for mental healthcare providers to be aware of the risks and benefits of psychedelic use, less than two-thirds felt they were sufficiently informed (64%) to discuss their patients’ personal use of psychedelics.

#### Group comparisons

Comparative analysis between groups showed no significant difference in self-rated knowledge.

#### Regression analysis

VIFs ranged from 4.735 to 6 due to high correlations between the levels of knowledge about the ‘benefits of psychedelics’ and the ‘benefits of psychedelics in a therapeutic context’ (*r* = 0.896, *p* < 0.001). Therefore, the variable ‘benefit of psychedelics’ was excluded from the model, which reduced the remaining VIFs to a range of 2 to 4.7. Self-rated knowledge of psychedelics significantly predicted actual knowledge about their current legal status in Australia (Nagelkerke *R*^2^ = 0.259, χ^2^(3) = 16.4, *p* < 0.001), suggesting that perceived knowledge of the risks, side effects and therapeutic benefits of psychedelics is associated with accurate knowledge of their legal status in Australia.

### Client interactions

#### Overall sample

Most clinicians (91.5%) indicated that they at least sometimes ask their clients about their use of psychedelics. While 58.6% of participants reported that their clients ask for advice about psychedelics for mental health treatment at least sometimes. Less than one-third (29.2%) of participants indicated that patients request preparation and/or integration psychotherapy for psychedelic use. However, nearly one-quarter (24.4%) of respondents indicated that they provide preparation and/or integration therapy for clients who have independently used psychedelic substances. Clinicians reported varying levels of comfort in discussing psychedelic use and addressing questions about its potential effects on clients, with 59.7% reporting feeling somewhat or very comfortable, with 15.9% feeling neutral and 24.4% feeling uncomfortable.

#### Group comparisons

Psychiatrists were significantly more likely than GPs to ask clients about their use of psychedelics (*t(79)* = 3.38, *p* = 0.003, Bonferroni corrected).

#### Regression analysis

Self-rated knowledge of psychedelics was not a significant predictor of willingness to provide preparation and/or integration therapy for clients who have taken psychedelic medicine independently ( $R_{adj}^{2}$ = 0.025, *F*(3,77) = 1.68, *p* = 0.179), suggesting that factors other than self-rated knowledge may play a more significant role in determining therapists’ willingness to provide such services.

### Attitudes toward psychedelics

#### Overall sample

**Figure 2.**
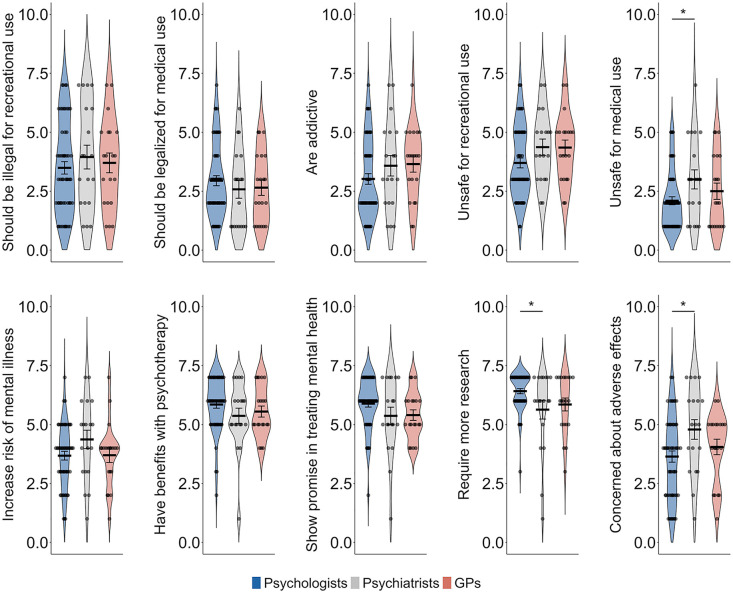
Attitudes toward psychedelics for recreational and medical use across mental health care provider groups. Violin plots display the distribution of responses for each group. Error bars represent the standard error of the mean. GP = general practitioner. *p < 0.05, **p < 0.01, ***p < 0.001.

Respondents had mixed attitudes on topics such as the legalization of psychedelics for recreational purposes (*M* = 3.63, *SD* = 1.95), the safety of recreational psychedelics use (*M* = 3.98, *SD* = 1.52), the potential of psychedelic use to increase the risk of subsequent psychiatric disorders (*M* = 3.83, *SD* = 1.44) and adverse mental health effects of psychedelics (*M* = 3.97, *SD* = 1.74). Over half of the participants (55.4%) did not perceive psychedelics to be addictive (*M* = 3.27, *SD* = 1.68). Most clinicians (71.7%) disagreed that psychedelics should be legalized for medical use without further clinical trials or research (*M* = 2.8, *SD* = 1.54). In addition, 78.3% agreed that psychedelics are safe even under medical supervision (*M* = 2.37, *SD* = 1.45). Furthermore, 91.1% agreed that psychedelics may improve outcomes when used in conjunction with psychotherapy (*M* = 5.68, *SD* = 1.19), 84.8% indicated that psychedelics shows promise in treating psychiatric disorders (*M* = 5.67, *SD* = 1.2) and 89.1% agreed that psychedelic warrant further research for the treatment of psychiatric disorders (*M* = 6.13, *SD* = 1.18), indicating strong support among clinicians for the supervised, clinical use of psychedelic-assisted therapy to treat mental illness (see Figure 2).

**Figure 3.**
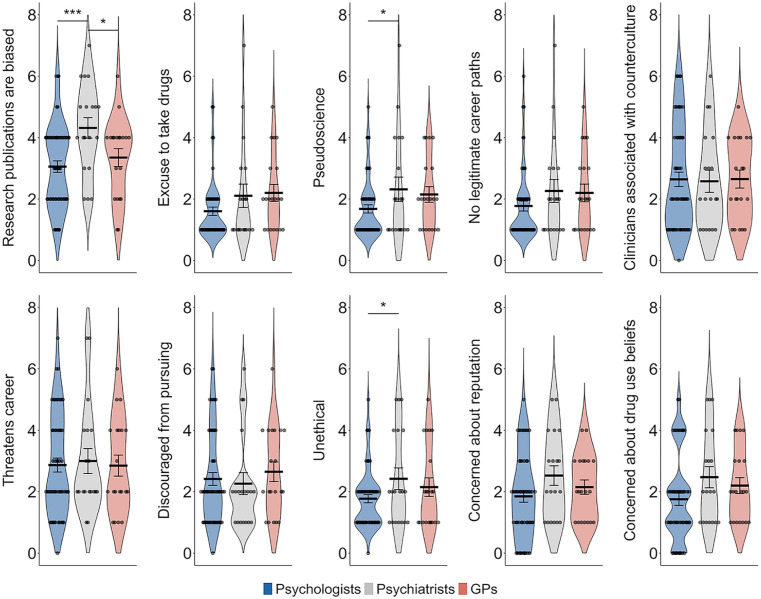
Stigmatizing attitudes and concerns about psychedelic-assisted therapy across mental health care provider groups. Error bars represent the standard error of the mean. GP = general practitioner. *p < 0.05, **p < 0.01, ***p < 0.001.

Clinicians generally refuted stigmatizing statements, with 85.9% disagreeing that psychedelic-assisted psychotherapy is pseudoscience (*M* = 1.91, *SD* = 1.22), 82.6% rejecting the notion that it is unethical (*M* = 1.99, *SD* = 1.21) and 88% disagreeing that it serves as an excuse for people to use drugs (*M* = 1.84, *SD* = 1.23). The majority supported psychedelic-assisted psychotherapy as a legitimate career path (87%, *M* = 1.97, *SD* = 1.31), did not associate it with counter-culture movements (68.1%, *M* = 2.63, *SD* = 1.58) or feel that supporting the field posed a threat to their professional career (64.8%, *M* = 2.89, *SD* = 1.63). Most clinicians also disagreed with statements suggesting they had been discouraged by colleagues or mentors from expressing an interest in psychedelic psychotherapy (72.5%, *M* = 2.43, *SD* = 1.49) and were not concerned about their professional reputation if they expressed an interest in this (60.9%, *M* = 2.05, *SD* = 1.33). They also disagreed that colleagues or clients would assume they use drugs if they practiced psychedelic medicine or psychedelic-assisted therapy (68%, *M* = 2.0, *SD* = 1.41). However, clinicians were more divided on the perception of scientific rigor in the field, with an average rating suggesting uncertainty (*M* = 3.38, *SD* = 1.44) and 37% choosing to neither agree nor disagree that published research on psychedelics is biased or lacks rigor (see Figure 3).

#### Group comparisons

Psychiatrists were significantly more likely than psychologists to perceive psychedelic use as unsafe even under medical supervision (*t(89)* = 2.38, *p* = 0.019, Bonferroni corrected) and to express concern about adverse mental health effects of psychedelic use (*t(89)* = 2.53, *p* = 0.013, Bonferroni corrected). Conversely, psychologists were significantly more likely than psychiatrists to agree that psychedelic use warrants further research for the treatment of psychiatric disorders (*t(89)* = 2.57, *p* = 0.012, Bonferroni corrected).

Psychiatrists were significantly more likely than psychologists to agree that published research on psychedelics is biased and lacks scientific rigor (*t(89)* = 3.45, *p* < 0.001, Bonferroni corrected) and were also more likely than GPs to share this view (*t(89)* = 2.21, *p* = 0.03, Bonferroni corrected). Psychiatrists were also more likely than psychologists to indicate that psychedelic medicine and psychedelic-assisted therapy are pseudoscience (*t(89)* = 1.98, *p* = 0.05, Bonferroni corrected) and to view the practices as unethical (*t(89)* = 2.03, *p* = 0.045, Bonferroni corrected).

### Access to information about psychedelics

The most common sources of information on psychedelic medicine and psychedelic-assisted therapy among clinicians were peer-reviewed journal articles (66%), news or magazines (digital or print, 47%), conferences (45%), podcasts (41%), Internet-based media like Wikipedia or Reddit (39%), patient experiences or case studies (35%) and webinars (34%). Additional sources included textbooks (20%), social media (17%), formal training programs (17%) and involvement in psychedelic research (16%). Overall, 82.6% of clinicians expressed interest in learning more about psychedelic-assisted psychotherapy. When asked about their preferred methods for further learning, clinicians indicated a preference for peer-reviewed journal articles (76%), conferences (74%) and formal training (67%). Fewer clinicians expressed interest in learning through webinars (58%), research involvement (57%) and patient involvement or case studies (52%).

### Experiences with psychedelics and other drugs

#### Overall sample

Approximately half (50.6%) of respondents reported never having used psychedelics (including psilocybin, LSD, DMT, 5-MeO-DMT, ayahuasca/acacia, mescaline and salvia). For those participants who reported past psychedelic use, the most commonly used psychedelics were psilocybin (40.9%) and LSD (40.9%). About one-quarter (23.9%) indicated they had microdosed at least once, with 65% reporting microdosing psilocybin, 30% LSD and 5% both psilocybin and LSD. Of those respondents who had experience with psychedelics, all but one (97.6%) reported positive experiences with these substances. Most clinicians agreed that psychedelics can enhance individuals’ connection to knowledge (89.6%), foster a sense of connection to others (86.3%) and facilitate mystical experiences (86.2%).

#### Group comparisons

Psychologists were significantly more likely than GPs to have used LSD (*t(85)* = 2.32, *p* = 0.023, Bonferroni corrected), cannabis (*t(73)* = 2.58, *p* = 0.012, Bonferroni corrected), ecstasy (*t(73)* = 2.05, *p* = 0.044, Bonferroni corrected) and MDMA (*t(73)* = 2.06, *p* = 0.043, Bonferroni corrected). In the survey, ecstasy and MDMA were listed separately, with ecstasy often referring to the street drug and MDMA to the pure compound.

#### Regression analysis

Personal experiences with psychedelics significantly predicted agreement with statements that psychedelics may improve outcomes when used in conjunction with psychotherapy ( $R_{adj}^{2}$ = 0.155, *F*(1,107) = 20.78, *p* = < 0.001) and that psychedelics show promise in treating psychiatric disorders ( $R_{adj}^{2}$ = 0.187, *F*(1,107) = 25.84, *p* < 0.001). Personal experiences also significantly predicted willingness to provide preparation and/or integration therapy for clients who have taken psychedelic medicine independently ( $R_{adj}^{2}$ = 0.065, *F*(1,80) = 6.56, *p* = 0.012). These results suggest that personal experiences may positively influence attitudes toward and openness to providing psychedelic-assisted therapy.

## Discussion

This cross-sectional study examined the attitudes, knowledge and experiences of Australian mental healthcare providers regarding psychedelics and psychedelic-assisted psychotherapy. Clinicians demonstrated positive attitudes toward psychedelic-assisted psychotherapy and its potential for treating psychiatric disorders, as well as support for continued research in the field. However, significant gaps in knowledge about the legal status, clinical indications and therapeutic applications of psychedelics were identified. Psychiatrists were more knowledgeable than GPs regarding the TGA approval but also expressed greater concerns about the validity of clinical trials, efficacy and safety. Clinicians’ self-rated knowledge correlated positively with actual knowledge. Many participants reported relying on non-academic sources for information. In line with previous studies (Meir et al., 2023; Žuljević et al., 2024), personal experience with psychedelics was linked to more positive attitudes toward psychedelic-assisted therapy and a greater willingness to provide support services such as preparation and integration therapy.

Our findings revealed generally positive attitudes among Australian mental healthcare providers toward the therapeutic use of psychedelics, consistent with previous research (Berger and Fitzgerald, 2023; Grover et al., 2023; Kucsera et al., 2023; Negrine et al., 2025; Wang et al., 2024; Wells et al., 2024). Strong support was expressed for further clinical trials, particularly those integrating psychotherapy into the therapeutic process. However, concerns about safety and limited efficacy data persisted, especially among psychiatrists. Psychiatrists were more likely than psychologists to view psychedelic use as unsafe, express concerns about potential adverse mental health effects and question the scientific rigor of current research. Some psychiatrists also perceived psychedelic-assisted therapy as pseudoscientific and unethical, while psychologists were generally more supportive of further research into the therapeutic potential of psychedelics for psychiatric disorders.

The knowledge base of clinicians regarding the legal status of psychedelics was mixed. While most correctly identified the TGA’s approval of MDMA for PTSD and psilocybin for treatment-resistant depression, fewer correctly identified the specific Schedule 8 classification. Interestingly, healthcare professionals were more likely to correctly identify psilocybin’s approval than MDMA, mirroring findings from a US-based study, where MDMA remained associated with recreational use and stigma (Wang et al., 2024). In a study by Wright et al. (2022), when randomly presented with identical clinical data supporting either MDMA-assisted psychotherapy or a neutrally labeled drug (i.e. JB-4801), psychiatrists were significantly less likely to recommend the MDMA trial to patients compared to psychologists and GPs (Wright et al., 2022). These findings underscore the need to disseminate accurate information to Australian clinicians to ensure safe and up-to-date practice. It also suggests persisting stigma and misinformation about MDMA, perhaps reflecting the broader cultural narratives around MDMA and its historical reputation as a ‘party drug’ (Garcya-Montes et al., 2021; Wolfgang et al., 2025; Wright et al., 2022).

Clinicians’ self-rated knowledge positively predicted their actual knowledge. This finding diverges from a recent health practitioner survey in the United States of America (Wang et al., 2024) and an Australian community survey (Nadeem et al., 2024), where an inverse relationship was found between self-perceived knowledge and actual knowledge. While this suggests a degree of accuracy in self-assessment among Australian clinicians, our findings also identified the need for further education, given that a substantial proportion still felt underinformed when discussing psychedelics with patients. Most healthcare providers reported at least sometimes asking clients about their psychedelic use, yet fewer offered preparation or integration support services. Notably, psychiatrists were more likely than GPs to inquire about psychedelic use. Previous research has found that patients are often reluctant to discuss psychedelic use with GPs due to concerns about ignorance and stigma (Boehnke et al., 2023), which may contribute to these patterns. With the growing mainstream attention on psychedelics, it is crucial that patients receive accurate information and mental healthcare providers facilitate this. Our findings emphasize the need for further education, particularly among Australian GPs, given their central role in referral pathways.

Peer-reviewed journal articles and conferences were the most frequently cited information sources. However, many clinicians also relied on informal channels like podcasts and Internet-based media, highlighting a gap in accessible, evidence-based information and training. Encouragingly, clinicians expressed the strongest interest in learning through journal articles, conferences and formal training programs, presenting an opportunity for professional organizations to provide structured education. Similarly, Wang et al. (2024) found that less than 10% of US healthcare professionals trusted pharmaceutical companies for information on psychedelics, preferring academic institutions, experienced clinicians and professional organizations (Wang et al., 2024). Interviews with Australian psychologists further revealed a strong desire for more education due to limited knowledge of psychedelic-assisted therapy (Negrine et al., 2025). Professional bodies such as the Royal Australian and New Zealand College of Psychiatrists (RANZCP) and the Australian Psychological Society (APS) could address this need by offering evidence-based training through journals, conferences and hands-on courses to equip mental healthcare providers for this emerging field.

Several limitations must be acknowledged. The sample was not representative of Australian mental health professionals, with an overrepresentation of psychologists, Queensland-based practitioners and individuals with previous psychedelic use, which was much higher than the Australian average (National Drug Strategy Household Survey 2022–2023, 2024). This bias may have influenced the positive attitudes observed, as previous use was associated with more favorable views (Wang et al., 2024). To reduce this kind of self-selection bias in future studies, psychedelic-focused questions should be embedded within broader surveys that assess attitudes toward a range of psychiatric treatments (e.g. antidepressants, TMS, ECT), rather than advertised as standalone psychedelic surveys, which may disproportionately attract individuals with pro-psychedelic views. Future research should aim for a broader national sample, explore potential generational differences between early-career and senior clinicians, and investigate the attitudes of psychiatry registrars versus consultants.

This study highlights generally positive attitudes among Australian mental healthcare providers toward psychedelic-assisted psychotherapy, coupled with significant gaps in knowledge and a reliance on informal information sources. While strong support exists for further research and the integration of psychedelics into mental health care, targeted educational initiatives are necessary to address safety concerns and knowledge gaps. Ensuring clinicians have access to accurate, evidence-based information will be essential in supporting the safe and effective implementation of psychedelic-assisted therapies in Australia.

## Supplemental Material

sj-docx-1-anp-10.1177_00048674251346679 – Supplemental material for Attitudes toward psychedelics and psychedelic-assisted psychotherapy among Australian mental healthcare providers Supplemental material, sj-docx-1-anp-10.1177_00048674251346679 for Attitudes toward psychedelics and psychedelic-assisted psychotherapy among Australian mental healthcare providers by Zohaib Nadeem, Stephen Parker, Hugh McGovern, Bianca Sebben and Lena KL Oestreich in Australian & New Zealand Journal of Psychiatry

sj-docx-2-anp-10.1177_00048674251346679 – Supplemental material for Attitudes toward psychedelics and psychedelic-assisted psychotherapy among Australian mental healthcare providers Supplemental material, sj-docx-2-anp-10.1177_00048674251346679 for Attitudes toward psychedelics and psychedelic-assisted psychotherapy among Australian mental healthcare providers by Zohaib Nadeem, Stephen Parker, Hugh McGovern, Bianca Sebben and Lena KL Oestreich in Australian & New Zealand Journal of Psychiatry
