# Supplementary material for: Attitudes toward psychedelics and psychedelic-assisted psychotherapy among Australian mental healthcare providers
Source: Aust N Z J Psychiatry. 2025 Jul 15;59(9):798–809. doi: 10.1177/00048674251346679 (PMC12397547; doi:10.1177/00048674251346679)
Supplement: sj-docx-2-anp-10.1177_00048674251346679 – Supplemental material for Attitudes toward psychedelics and psychedelic-assisted psychotherapy among Australian mental healthcare providers [file sj-docx-2-anp-10.1177_00048674251346679.docx]

**Supplementary Material 2: Survey questions adapted for the Australian context from Kucsera et al. (2023)**

For the purpose of this research, the term “psychedelic” will refer to classic hallucinogens including lysergic acid diethylamide (LSD), psilocybin, dimethyltryptamine (DMT), ayahuasca, and mescaline, as well as methylenedioxymethamphetamine (MDMA) but NOT KETAMINE or CANNABIS.

Please answer the following questions as honestly as possible without looking up the answers. We are interested in your baseline knowledge, so a “best guess” is fine for this survey.

In Australia, at the federal level, psychedelic use is currently

- Illegal
- Legal
- Legal for medical use only
- Decriminalized
- Don't know

In Australia, psychedelics are currently classified by the Therapeutic Goods Administration (TGA) as __________ drugs.

- Schedule 1 (Not currently in use)
- Schedule 2 (Pharmacy Medicine)
- Schedule 3 (Pharmacist Only Medicine)
- Schedule 4 (Prescription Only Medicine OR Prescription Animal Remedy)
- Schedule 5 (Caution)
- Schedule 6 (Poison)
- Schedule 7 (Dangerous Poison)
- Schedule 8 (Controlled Drug)
- Schedule 9 (Prohibited Substance)
- Schedule 10 (Substances of such danger to health as to warrant prohibition of sale, supply and use)
- Unscheduled
- Don’t know

The TGA has provided conditional approval for the prescription of MDMA in the management of which of the following mental disorders:

- Bipolar disorder
- Treatment-resistant depression
- Posttraumatic stress disorder
- Generalized anxiety disorder
- Don’t know

The TGA has provided conditional approval for the prescription of psilocybin in the management of which of the following mental disorders:

- Bipolar disorder
- Treatment-resistant depression
- Posttraumatic stress disorder
- Generalized anxiety disorder
- Don’t know

Rate your level of knowledge about (rating scale from 0 – none to 10 – high)

- The side effects of psychedelic use
- The risks of psychedelic use
- The potential benefits of psychedelic use
- The potential therapeutic benefits of psychedelics in a therapeutic context

How important is it for mental healthcare providers to be aware of the risks and benefits of psychedelic use?

- Very important
- Somewhat important
- Neutral
- Somewhat unimportant
- Very unimportant

Please indicate the extent to which you agree or disagree with the following (rating scale from 1 – strongly disagree to 7 – strongly agree)

- Psychedelics should be illegal for recreational purposes
- Psychedelics should be legalized for medical use without further clinical trials or research
- Psychedelic substances are addictive
- Psychedelic use is unsafe when taken recreationally
- The use of psychedelics is unsafe even under medical supervision
- Psychedelic use increases the risk of subsequent psychiatric disorders
- Psychedelic use may improve outcomes when used in conjunction with psychotherapy
- Psychedelic use shows promise in treating psychiatric disorders
- Psychedelic use warrants further research for the treatment of psychiatric disorders
- I am concerned about the adverse mental health effects of psychedelic use

How informed are you to talk to patients/clients about their personal use of psychedelics?

- Very informed
- Somewhat informed
- Neutral
- Somewhat uninformed
- Very uninformed

Are you a practicing clinician?

- No
- Yes

I ask my patients/clients about their use of psychedelics

- Always
- Most of the time
- About half the time
- Sometimes
- Never

My patients/clients ask me for advice about psychedelics for the treatment of mental health issues (e.g., microdosing, interactions with other medications, risks/benefits, information about clinical trials).

- Always
- Most of the time
- About half the time
- Sometimes
- Never

My patients/clients ask me to provide preparation and/or integration psychotherapy surrounding their own psychedelic use (either legal or illegal).

- Always
- Most of the time
- About half the time
- Sometimes
- Never

I provide preparation and/or integration therapy for my patients/clients who have taken psychedelic medicine independently (either legally or illegally).

- Agree
- Most of the time
- About half the time
- Sometimes
- Never

How comfortable are you talking to patients/clients about psychedelic use?

- Very comfortable
- Somewhat comfortable
- Neutral
- Somewhat uncomfortable
- Very uncomfortable

How comfortable are you addressing patient questions about how psychedelics might affect them (e.g., risks, benefits, side effects, medication interactions)?

- Very comfortable
- Somewhat comfortable
- Neutral
- Somewhat uncomfortable
- Very uncomfortable

Please indicate to what extent you agree or disagree with the following (rating scale from 1 – strongly disagree to 7 – strongly agree)

- The published research on psychedelics is biased and lacks scientific rigor
- Psychedelic-assisted psychotherapy is just an excuse for people to take drugs
- Psychedelic medicine and psychedelic-assisted psychotherapy is pseudoscience
- Psychedelic medicine and psychedelic-assisted psychotherapy are not legitimate career paths
- Clinicians associated with psychedelic medicine are part of the counterculture
- Expressing a professional interest in psychedelic medicine or psychedelic assisted would threaten my career (e.g., finding a job, career growth)
- I have been discouraged from expressing interest in psychedelic medicine or psychedelic assisted psychotherapy by mentors or colleagues
- Clinicians who offer psychedelic-assisted therapy (e.g., preparation, integration, harm-reduction) have unethical practices

Please indicate to what extent you are concerned or unconcerned with the following (rating scale from 1 – very unconcerned to 5 – very concerned)

- To what degree would you be concerned about your professional reputation if you expressed a professional interest in psychedelic medicine or psychedelic assisted psychotherapy?
- To what degree would you be concerned that colleagues or clients would think you used drugs if you practiced psychedelic medicine or psychedelic assisted psychotherapy?

Please answer the following about sources of information.

From which of the following sources have you obtained information on psychedelic medicine and psychedelic-assisted therapy (select all that apply)?

- Peer-reviewed journal article(s)
- Textbook(s)
- Conference (in person or virtual)
- Patient experience or case study
- Psychedelic research involvement
- Webinar
- News or magazine article (digital or print)
- Internet-based media (e.g., Wikipedia, Reddit, blog)
- Podcast(s)
- Social media (e.g., Facebook, Instagram, etc.)
- Formal training program (e.g., MAPS, CIIS)
- Other, please specify _________________________
- None

I'd be interested in learning more about psychedelic medicine and/or psychedelic-assisted psychotherapy via (select all that apply):

- Peer-reviewed journal article(s)
- Textbook(s)
- Conference (in person or virtual)
- Patient experience or case study
- Psychedelic research involvement
- Webinar
- News or magazine article (digital or print)
- Internet-based media (e.g., Wikipedia, Reddit, blog)
- Podcast(s)
- Social media (e.g., Facebook, Instagram, etc.)
- Formal training program (e.g., MAPS, CIIS)
- Other, please specify _________________________
- None

How interested are you in learning more about psychedelic medicine and/or psychedelic-assisted psychotherapy?

- Very uninterested
- Somewhat uninterested
- Neither interested nor uninterested
- Somewhat interested
- Very interested

Any additional comments, concerns, opinions on psychedelics that you'd like to share?

________________________________________________________________

**Original article:** Kucsera A, Suppes T, Haug NA. Psychologists’ and psychotherapists’ knowledge, attitudes, and clinical practices regarding the therapeutic use of psychedelics. Clin Psychol Psychother. 2023;30(6):1369–79
